# Proximity Ligation Assay: From a Foundational Principle to a Versatile Platform for Molecular and Translational Research

**DOI:** 10.3390/biom15101468

**Published:** 2025-10-17

**Authors:** Hengxuan Li, Xiangqi Ma, Dawei Shi, Peng Wang

**Affiliations:** 1 Medical School, Faculty of Medicine, Tianjin University, Tianjin 300072, China; tjdxlhx@tju.edu.cn; 2 Hangzhou Institute of Medicine (HIM), Chinese Academy of Sciences, Hangzhou 310022, China; yc17659@um.edu.mo; 3 National Institutes for Food and Drug Control, Beijing 100050, China; 4 Faculty of Health Sciences, University of Macau, Macau SAR 999078, China

**Keywords:** proximity ligation assay, protein–protein interactions, biomarkers, infectious diseases diagnostic

## Abstract

The precise analysis of protein interactions in their native cellular context and the sensitive quantification of protein abundance in biological fluids are both fundamental to understanding health and disease. Traditional methods for cellular imaging and biochemical quantification often face limitations in specificity, sensitivity, or the preservation of spatial information. The proximity ligation assay (PLA) is a versatile technological platform developed to overcome these challenges by converting protein recognition events into amplifiable DNA signals, thereby achieving exceptional sensitivity. This foundational principle has given rise to two major formats: in situ PLA (isPLA) and solution-phase PLA. In basic research, isPLA provides high-resolution visualization of protein–protein interactions (PPIs), post-translational modifications (PTMs), and subcellular architecture directly within fixed cells and tissues. In translational and clinical applications, solution-phase PLA enables the highly sensitive quantification of low-abundance biomarkers in liquid samples, which is critical for diagnostics and prognostics in fields such as oncology, neuroscience, and infectious diseases. This review discusses the foundational principles, development, and diverse applications of PLA platforms. We also highlight significant technological advancements, including the development of high-throughput formats, integration with advanced readouts, and the use of alternative affinity reagents. These innovations continue to transform PLA from a targeted validation method into a powerful and multifaceted platform for both fundamental systems biology and clinical diagnostics.

## 1. Introduction

Studies on protein interactions and modifications within cells and tissues can deepen the understanding of various fields, from normal cellular functions to disease-related disorders [1,2]. In the past, researchers have employed various methods to study these molecular events [3,4,5]. However, traditional methods face a fundamental conflict between the accuracy of biochemical analysis and the preservation of spatial information. Biochemical methods identify protein–protein interactions (PPIs) by separating protein complexes from cell lysates [6]. The main limitation of these methods lies in the requirement for cell lysis, a process that destroys the original cellular structure, leading to the loss of spatial and temporal information about intracellular PPIs [7]. In contrast, imaging techniques such as Förster resonance energy transfer (FRET) and bimolecular fluorescence complementation (BiFC) enable the visualization of PPIs within intact cells [8,9,10]. However, these methods also have limitations regarding the overexpression of exogenous fusion proteins. Overexpression of labeled proteins may cause environmental distortion, mislocalization, and steric hindrance, which may affect the biological relevance of PPIs [2,11]. In parallel with the challenges in cellular imaging, a distinct set of limitations was associated with the quantitative measurement of proteins in biological fluids [12]. For decades, the enzyme-linked immunosorbent assay (ELISA) has been the standard method for protein quantification in samples such as plasma and serum [13]. However, ELISA often lacks the sensitivity required to detect low-abundance protein biomarkers, which are critical for the early diagnosis and monitoring of diseases [14].

Therefore, it is important to develop a new technique to overcome the limitations of traditional methods; this technique can analyze endogenous proteins with high sensitivity and specificity. The proximity ligation assay (PLA) combines the high specificity of biochemical methods with the high spatial resolution of imaging techniques, enabling highly sensitive and specific analysis [15]. The principle of PLA is that a quantifiable signal is generated only when two or more antibody–oligonucleotide conjugates bind target epitopes that are in proximity, allowing the attached oligonucleotide to be joined by ligation [16]. Based on proximity ligation principle, PLA has evolved into two major, complementary platforms: in situ PLA (isPLA) and solution-phase PLA. isPLA serves as an effective indicator of protein co-localization, bridging the gap between biochemical detection and cellular imaging [17,18]; solution-phase PLA was adapted for the highly sensitive quantification of proteins in biological fluids, where it can detect low-abundance biomarkers for applications in oncology, neuroscience, and infectious diseases [19,20,21].

**Figure 1:**
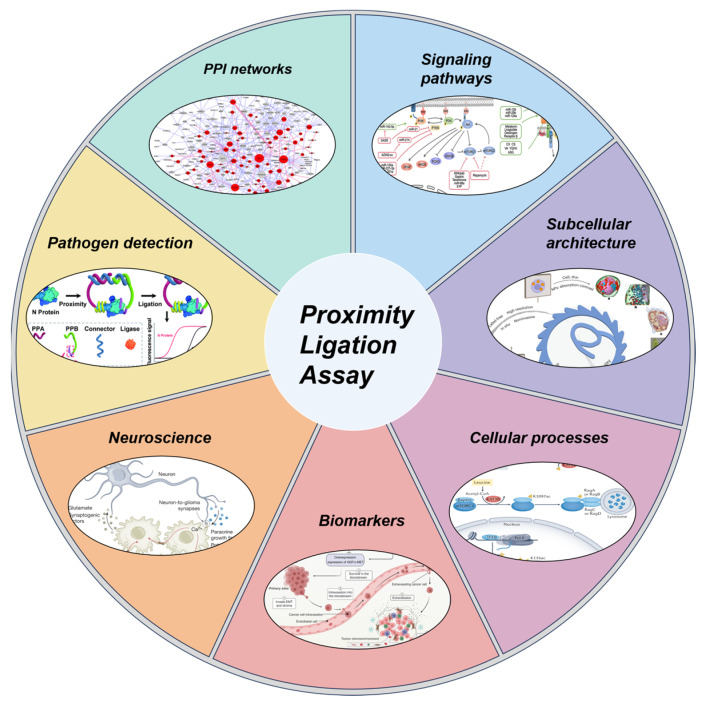
Applications of PLA across different fields.

As a highly sensitive and specific molecular detection technique, the application of PLA has expanded from PPIs research to multiple fields in basic biology and clinical medicine (Figure 1). In basic cell biology research, the most widespread application of isPLA is the validation and visualization of PPIs, overcoming the limitations of traditional methods which often require protein overexpression [22]. The core advantage of isPLA lies in its ability to perform in situ detection and quantification of endogenous molecular events in cell or tissue samples, thereby providing valuable spatiotemporal information. Researchers utilized isPLA to precisely localize specific protein complexes at the subcellular level and determined their presence in specific regions such as mitochondria, the nucleus, or the cell membrane [17]. Additionally, isPLA is widely used for detecting post-translational modifications (PTMs) of proteins. By employing specific antibodies specific to the target protein, isPLA can in situ detect the modification status of specific proteins, which is crucial for studying dynamic cellular processes [23,24,25]. In the fields of translational medicine and clinical applications, PLA demonstrates significant potential as a powerful tool for diagnostic and prognostic biomarkers. In neuroscience, the high sensitivity of solution-phase PLA enables the detection of low-abundance pathological protein oligomers, such as Aβ protofibrils and α-synuclein aggregates. Since these oligomers appear in the early stages of neurodegenerative disorders like Alzheimer’s disease (AD) and Parkinson’s disease (PD), solution-phase PLA offers new opportunities for early diagnosis [26,27]. The application of solution-phase PLA in pathogen detection and infectious disease diagnosis is also expanding; it enables high-sensitivity diagnosis of viral antigens or bacterial toxins, thereby revealing the molecular mechanisms of infection and guiding clinical treatment [16,28,29]. In summary, with the capabilities for in situ analysis, high sensitivity, and quantitative detection, PLA has become a crucial bridge connecting fundamental molecular biology research with clinical diagnostic applications.

## 2. The Proximity Ligation Assay

### 2.1. The Principle of PLA

PLA is a powerful and versatile technological platform that translates the detection of proteins and their molecular interactions into an amplifiable nucleic acid signal, thereby achieving exceptional sensitivity and specificity [30,31]. The principle of PLA depends on a proximity-dependent enzymatic reaction. When two proximity probes bind to either different epitopes on the same target protein or to two separate proteins within a complex, the DNA oligonucleotides attached to them are brought into close physical proximity (generally less than 40 nanometers). After a dilution step to reduce background, this colocalization allows oligonucleotides on antibody–oligonucleotide conjugates to hybridize to the same connector oligonucleotide, guiding their joining by ligation to create a new DNA molecule. The newly formed DNA strand serves as an amplifiable surrogate marker, where its presence and quantity report on the presence and quantity of the initial target protein or protein complex [32]. This core principle has been adapted into two major distinct modalities that address different scientific questions.

**Figure 2:**
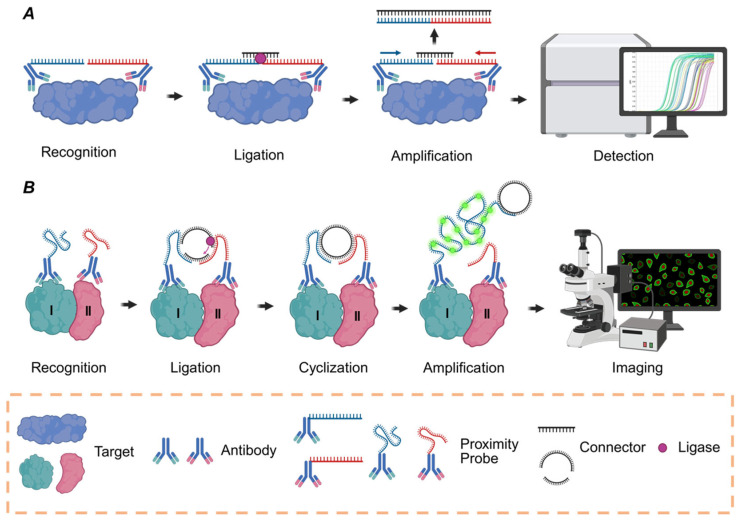
Schematic of the PLA principle. (**A**) Schematic illustration of solution-phase PLA. (**B**) Schematic illustration of isPLA.

The first developed format for PLA is solution-phase PLA (Figure 2A). In this method, proximity probes are first incubated with a sample to allow for binding to the target protein. When the probes bind to adjacent sites on the target, a connector oligonucleotide is introduced, which hybridizes to the single-stranded DNA (ssDNA) tags on each probe. A DNA ligase then joins the tags to form a new DNA molecule that serves as a template for amplification and quantification by qPCR. Solution-phase PLA is a quantitative method for analyzing proteins in biological fluids such as plasma and serum. This approach provides sensitive and high-throughput measurements of protein concentrations, making it a tool for applications such as biomarker discovery and validation [33,34].

In situ PLA (isPLA) is a visualization technique applied to fixed cells and tissues (Figure 2B). In this method, a target is first recognized by pairs of oligonucleotide-conjugated antibodies. After washes, added pairs of oligonucleotides can be ligated to form DNA circles, guided by proximal pairs of antibody–oligonucleotide conjugates. The circular DNA molecule serves as a template for rolling circle amplification (RCA). The RCA process generates a long ssDNA product composed of tandem repeats of the circular template. The product of RCA is then detected through the hybridization of fluorescently labeled oligonucleotide probes [35]. This method enables individual proteins, PPIs, and PTMs to be visualized, localized, and semi-quantified within the native cellular context. The ability of isPLA to provide spatial information makes it a valuable tool for validating molecular interactions initially identified by non-spatial, high-throughput methods [17].

In conclusion, instead of being a single technique, PLA is a foundational principle that has given rise to two distinct and complementary technological platforms. isPLA allows researchers to visualize molecular events, such as protein expression, interactions, and modifications, within their native spatial context, serving as a key tool for mechanistic cell biology, pathology, and for validating molecular hypotheses directly within cells and tissues. Solution-phase PLA enables the sensitive, specific, and high-throughput measurement of protein concentrations in systemic biofluids by coupling the proximity principle with the quantitative power of PCR, making the assay a primary tool for clinical proteomics, biomarker discovery, and systems biology. A detailed comparison between the two methods is presented in Table 1.

### 2.2. The Development History of PLA

**Figure 3:**
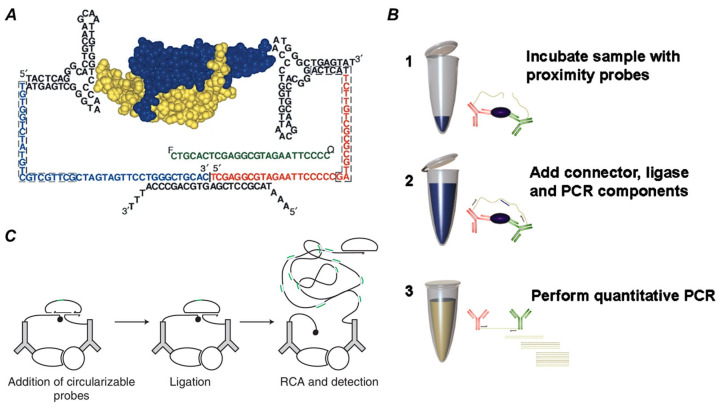
The development history of PLA. (**A**) Schematic view of the homodimeric PDGF-BB bound by two aptamer-based proximity probes for detection by solution-phase PLA. Reprinted/adapted with permission from Ref. [36]. Copyright 2002, Springer Nature. (**B**) The principal steps of antibody-based solution-phase PLA for the detection of various cytokines. Reprinted/adapted with permission from Ref. [37]. Copyright 2004, National Academy of Sciences. (**C**) Detection of endogenous c-Myc/Max heterodimers in cultured cells using isPLA [35]. Reprinted/adapted with permission from Ref. [35]. Copyright 2006, Springer Nature.

In 2002, Landegren et al. established the conceptual foundation for solution-phase PLA. Their pioneering work introduced a novel principle for protein detection that involved converting a protein recognition event into a unique, amplifiable DNA molecule (Figure 3A). This group used pairs of DNA aptamers targeting the cytokine platelet-derived growth factor (PDGF). Aptamers are short single-stranded nucleic acids that binds to target proteins using specific three-dimensional conformations. Two aptamers that recognized different epitopes on PDGF were each conjugated to an oligonucleotide, forming a pair of proximity probes. When the pair of probes bound to the same PDGF molecule simultaneously, their attached oligonucleotide were pulled closer together. This allowed the oligonucleotides to be connected via DNA ligase with the assistance of the connector, forming a new DNA sequence. The target-dependent DNA products were quantified by qPCR. Ultimately, this detection method achieved a limit of detection (LOD) for PDGF of 40 × 10^−21^ mol, representing approximately a 1000-fold increase in sensitivity compared to traditional ELISA [36].

Recognizing the enormous potential of PLA, the technology was quickly extended to utilize antibodies as proximity ligation probes. In 2004, Gullberg et al. demonstrated antibody-based solution-phase PLA for the detection of various cytokines, establishing a versatile and applicable platform (Figure 3B). This prompted researchers to apply existing antibodies to the study of a variety of proteins [37].

In 2006, Landegren et al. applied PLA to in situ analysis, and this innovative technology, termed isPLA, achieved the direct visualization of individual endogenous protein complexes at the single-molecule level in fixed cell and tissue samples. In this work, they visualized the interaction between the oncogenic transcription factors Myc and Max in the cell nucleus (Figure 3C). Experimental results also showed that the frequency of the interaction could be modulated by signal cues and small-molecule inhibitors. This work established isPLA as an important imaging tool in cell biology, providing a method for studying the spatial information of PPIs in the native environment. Subsequently, the commercialization of isPLA enabled its broader application in biomedical research [35].

### 2.3. The Classification of PLA

To accommodate different experimental requirements, PLA has been developed into multiple forms, all of which are based on the core principle of proximity ligation.

isPLA is the most common form of PLA, performed on fixed cells or tissue sections on microscope slides [35]. The primary advantage of isPLA is the preservation of the spatial environment, allowing researchers to visualize the presence and precise subcellular localization of PPIs [38]. This method is valuable for studying the structure of signaling complexes and is compatible with standard immunofluorescence (IF) and immunohistochemistry (IHC), including the analysis of formalin-fixed paraffin-embedded (FFPE) tissues [39].

Flow-PLA is a variant designed for the analysis of single cells or subcellular particles in suspension. With this approach, protein interactions and modifications are detected within individual entities such as cells, extracellular vesicles, or synaptosomes [40,41]. Both internal and superficial molecular events can be captured. By coupling the isPLA protocol with flow-based analysis platforms like flow cytometry or mass cytometry, quantitative and high-throughput analysis of complex samples is enabled [42]. Furthermore, the application of the technique in neuroscience for analyzing protein complexes in synaptosomes highlights the utility of the method in specialized research fields [43].

In contrast to isPLA, solution-phase PLA is suitable for analyzing liquid samples such as serum, plasma, and cell lysates, with the experiment usually carried out in microtiter plates. The core of solution-phase PLA is the homogenous mode, where the sample, proximity probes, and enzymatic reagents are mixed directly in a single reaction volume [44]. This wash-free process not only simplifies the procedure but also allows for easy integration into high-throughput automation platforms. However, the lack of washing prevents the removal of components from the biological matrix, which may inhibit the enzymatic reactions [16,37].

The Proximity Extension Assay (PEA) is another prominent solution-phase technology that shares the dual-recognition principle with PLA but employs a distinct enzymatic mechanism. In the PEA method, a DNA polymerase is utilized to extend one probe along the other, which serves as a template, rather than using DNA ligase to join two probes [45]. This mechanistic difference makes the assay format particularly well-suited for highly multiplexed, quantitative protein measurements. The primary application of PEA is in large-scale biomarker discovery and proteomics screening from liquid samples [46,47]. Commercial platforms Olink Explore based on PEA can be used to simultaneously quantify thousands of different proteins in samples like plasma and serum [48,49,50]. While both assays are based on molecular proximity, the applications of PLA and PEA often differ. PLA, particularly isPLA, is used primarily to visualize and localize specific protein interactions within the cellular context. In contrast, PEA is optimized for the high-throughput, sensitive quantification of protein abundance in bulk liquid samples, where spatial information is exchanged for broader screening capabilities [51].

Solid-phase PLA is a method that combines the sandwich immunoassay and solution-phase PLA technologies, effectively enhancing specificity and resisting interference from sample matrices [19]. At the start of the solid-phase PLA process, capture antibodies are immobilized on a solid-phase support to bind to the target protein in the sample. After a single wash, unbound components and interferences are removed. Followed by another wash, proximity probes are added, and the ligation and amplification reactions are performed. Due to the use of a solid-phase format, this method can process larger sample volumes and concentrate target molecules, thereby enhancing sensitivity. Additionally, these washing steps significantly reduce background signals, making solid-phase PLA particularly suitable for detecting biomarkers in complex samples such as plasma [44].

### 2.4. Comparison Between isPLA and Key PPI Methods

To highlight the unique advantages of isPLA for studying PPIs, it is necessary to compare it with other methods. Each method has its own advantages and disadvantages, and the choice of method depends on the specific research question. A comparison between isPLA and other key PPIs methods is shown in Table 2.

Immunoprecipitation (Co-IP) is considered the gold standard for validating PPIs. Co-IP uses antibodies to separate target proteins from cell lysates, which are then identified by Western blotting. The core advantage of Co-IP is that it can effectively confirm the interactions between proteins between endogenous proteins within cells [52].

In contrast, isPLA exhibits several unique advantages. isPLA is an in situ detection technique that preserves the integrity of cellular structures during operation. As a result, isPLA retains the specific location information of interactions occurring within cells [53]. isPLA has higher detection sensitivity and milder reaction conditions. This enables it to capture weak and transient interactions. In contrast, such signals are easily lost during the stringent cell lysis and washing steps of Co-IP [54]. isPLA requires a smaller initial sample size, making it suitable for analyzing precious materials such as clinical biopsy samples [16].

The principle of FRET is that when donor and acceptor fluorescent molecules are in close proximity (typically 1–10 nanometers), energy transfer can occur, and can be detected to determine the degree of proximity between molecules [55]. The core advantage of FRET is the ability to monitor molecular interactions within living cells in real time and dynamically with high spatial resolution [56]. Compared to FRET, isPLA can directly analyze endogenous, unmodified target proteins [53,57]. FRET requires the fusion expression of target proteins with fluorescent reporter proteins like GFP or YFP. However, this exogenous labeling strategy may induce non-physiological artifacts due to protein overexpression, such as misvocalization or functional disruption [58,59]. Although isPLA has a simpler workflow and more reliable results, the ability of FRET to monitor the dynamics of interactions in live cells in real time is irreplaceable [60,61].

The yeast two-hybrid (Y2H) system is a powerful genetic method for discovering novel PPIs through large-scale screening. In this system, two target proteins are fused to the DNA-binding domain (BD) and activation domain (AD) of the transcription factor. If target proteins interact, the separated BD and AD will reconstitute a fully functional transcription factor due to their spatial proximity, thereby initiating the transcription of the downstream reporter gene [62]. However, Y2H has a high rate of false positives and false negatives. The detection environment for PPIs is limited to the yeast cell nucleus, and this heterologous environment may not fully mimic the actual physiological conditions in mammalian cells, such as the absence of species-specific cofactors or necessary PTMs [63]. Therefore, isPLA serves as a critical next validation technique. It can effectively validate the potential interactions identified by Y2H in the target mammalian cell type and the correct subcellular compartment [64]. Y2H and isPLA are highly complementary in research strategies, Y2H primarily is a high-throughput screening tool for initial discovery, while isPLA provides precise, targeted validation of these discoveries in a physiologically relevant context.

## 3. Foundational Applications of isPLA in Cellular Biology

### 3.1. Mapping PPI Networks In Situ

In basic research, the core application of isPLA is the visualization and in situ validation of PPIs in natural cellular environments [65]. isPLA can effectively confirm whether spatial proximity exists between proteins. Compared to interactions inferred from colocalization data, the close physical proximity provides more conclusive evidence for PPIs [53]. Additionally, isPLA can detect both stable and transient interactions in endogenous proteins while avoiding false positives introduced by protein overexpression systems [66].

In the initial PLA technology, isPLA demonstrated the application value in PPIs research. This pioneering study successfully visualized the interaction between the endogenous transcription factors Myc and Max. The study confirmed the known interaction of Myc and Max within the nucleus, and showed that the frequency of the interaction could be dynamically modulated by interferon-gamma signaling or small-molecule inhibitors. This result demonstrated the potential of isPLA to probe the regulation of PPIs [35,67]. The foundational work paved the way for numerous subsequent studies in the field of cell biology [68]. Furthermore, isPLA has been widely used to confirm structural interactions, such as the association between E-cadherin and β-catenin at adherens junctions, a process fundamental to epithelial tissue integrity [69]. It has also been applied to map interactions within specific compartments, like the association of podocalyxin and ezrin in the membrane structures of renal glomeruli [70].

**Figure 4:**
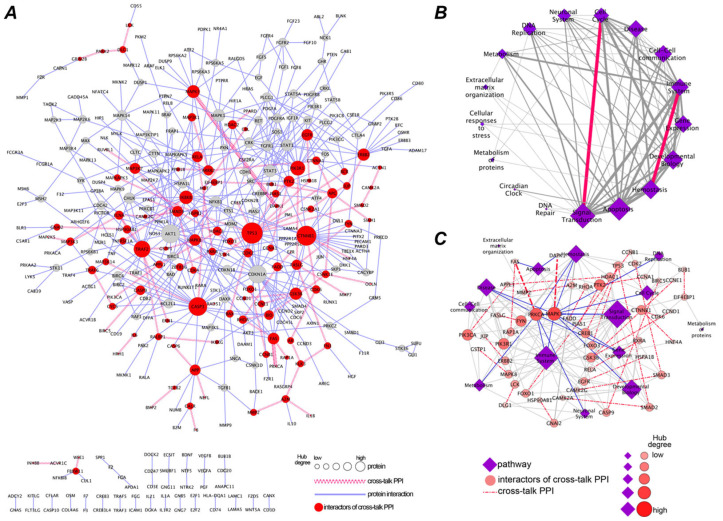
Using an isPLA to systematically profile endogenous protein–protein interactions in a Pathway Network. Reprinted/adapted with permission from Ref. [71]. Copyright 2014, American Chemical Society. (**A**) An architectural map of the network comprising the 557 endogenous PPIs. (**B**) The pathway–pathway interaction network by 90 cross-talk PPIs. (**C**) The cross-talk PPI and pathway interaction network.

Initially, isPLA was primarily used to validate single, hypothetical interactions. However, with the development of isPLA, its application has shifted from testing hypotheses to proposing hypotheses. Researchers systematically map the local interaction networks of single targets by screening antibody libraries. For network data generated by non-spatially resolved techniques such as affinity purification-mass spectrometry (AP-MS), isPLA can supplement them with critical cellular and spatial dimensional information. For example, Hung et al. used isPLA to analyze 1204 endogenous PPIs in the HeLa cell signaling networks, which validated 557 interactions and identified 8 new ones (Figure 4). This work also showed the ability of isPLA to identify “cross-talk” PPIs connecting different signaling pathways, such as the interaction between MAPK3 and DAPK1, which connects 9 distinct pathways. The capacity to construct and validate spatially resolved protein interaction networks makes isPLA an essential tool for systems biology, connecting large-scale interactome data with the functional organization of proteins in the cell [71].

### 3.2. Elucidating PTMs and Signaling Cascades

The function and state of proteins are precisely regulated by PTMs [72]. Modifications like phosphorylation, ubiquitination, and SUMOylation act as molecular switches, controlling protein activity, stability, and interactions [73]. isPLA can detect specific PTMs by combining two antibodies targeting the same protein. isPLA offers a unique approach to visualize specific protein subpopulations at single-molecule resolution under specific functional states and provides precise subcellular localization information.

**Figure 5:**
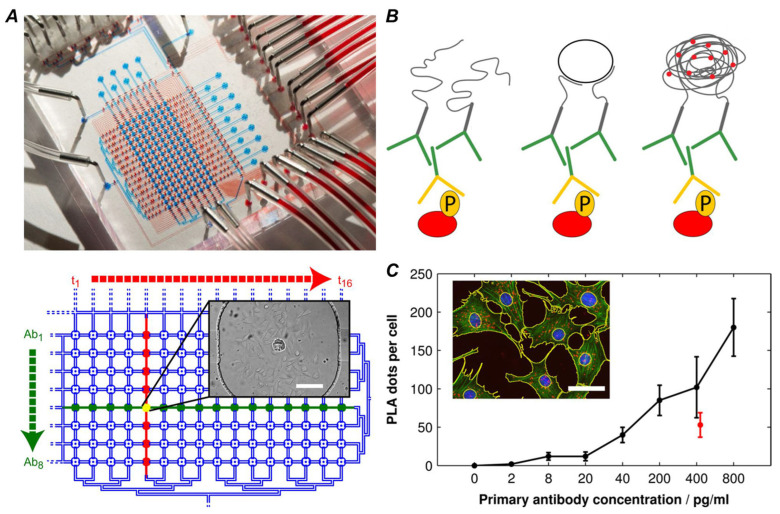
isPLA for high-content profiling of cell signaling pathways on a microfluidic chip Reprinted/adapted with permission from Ref. [77]. Copyright 2013, Elsevier. (**A**) Large-scale integrated microfluidic chip. (**B**) The working principle of the integrated isPLA. (**C**) On-chip PLA with varying anti-Akt primary antibody concentrations.

Visualization of protein phosphorylation states is a core application of PTM-PLA, as it directly reflects kinase activity and the propagation of signals through intracellular cascading pathways. This method is widely used to monitor the activation of cell surface receptor tyrosine kinases (RTKs) [74]. For example, using isPLA to detect the phosphorylation levels of EGFR or PDGFR-β after ligand stimulation provides a more direct assessment of receptor activation compared to measuring total receptor protein levels [75]. isPLA is also applicable for detecting downstream signaling proteins; studies have used isPLA to track the phosphorylation of key kinases such as Akt and ERK, thereby mapping the spatiotemporal dynamics of information flow within pathways like PI3K/Akt/mTOR [76]. The integration of isPLA with microfluidic technology enables high-content, dynamic analysis of these signaling pathways and simultaneous measurement of the activation kinetics of multiple phosphorylation events. A study using a microfluidic isPLA chip precisely characterized the activation features of the Akt signaling pathway under PDGF stimulation by monitoring the phosphorylation of Akt, GSK-3β, and S6, and determined that the maximum activation time for Akt and its direct substrates was 4 to 8 min (Figure 5) [77].

Additionally, isPLA is a powerful tool for studying ubiquitin- and SUMO-mediated regulatory events. Ubiquitination is a key signal for protein degradation, DNA repair, and signal transduction [78]. isPLA has been used to visualize target protein ubiquitination after treatment with proteolysis-targeting chimeras (PROTACs). This application provides direct in situ evidence of the mechanism of action by demonstrating the formation of a ternary complex involving the target protein, PROTAC, and E3 ligase, and the subsequent ubiquitination [79]. In studies of DNA damage response, isPLA has been used to demonstrate that the FEN1 protein is modified by SUMO-1 following UV irradiation. The results showed that the SUMOylation modification promoted the interaction between FEN1 and the Rad9-Rad1-Hus1 checkpoint complex, enhancing its function in repairing stalled replication forks. These works demonstrate the capabilities of isPLA, which is not merely a measure of protein abundance but can visualize the active functional state of the proteome, revealing how PTMs regulate complex cellular activities in specific subcellular regions [80].

### 3.3. Investigating Subcellular Architecture and Organelle Crosstalk

The eukaryotic cell is a highly structured space in which function and structure are closely interconnected. Organelles do not exist in isolation; they communicate dynamically via membrane contact sites (MCS). These are specialized regions where the membranes of two organelles are in close proximity to exchange lipids, ions, and signaling molecules [81]. Since the isPLA detection range of below 40 nm corresponds to MCS sizes, the assay has become a valuable tool for measuring inter-organelle contacts.

The most studied example is the mitochondria-ER contact sites (MERCS) between the endoplasmic reticulum (ER) and mitochondria. These sites are key platforms for calcium homeostasis, lipid metabolism, and apoptosis regulation [82]. isPLA is commonly utilized to measure MERCS by monitoring the closeness of ER-resident proteins, like VAPB or IP3R, to outer mitochondrial membrane proteins, such as PTPIP51 or VDAC [83]. This method has been applied to examine how MERCS are modified in disease models. For instance, isPLA was employed in AD post-mortem brain tissue to measure the VAPB-PTPIP51 interaction, providing insight into defective ER-mitochondria communication in neurodegeneration [84].

**Figure 6:**
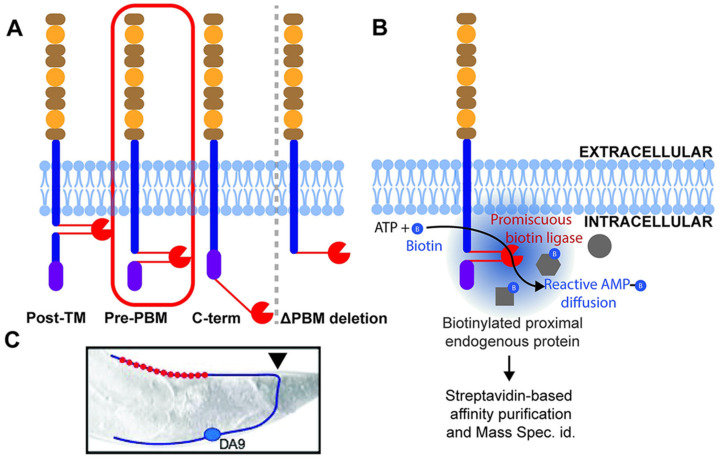
Characterization of the intracellular neurexin interactome by in vivo proximity ligation. Reprinted/adapted with permission from Ref. [86]. Copyright 2024, Public Library of Science. (**A**,**B**) Schematic of the rationale and workflow for the proteomics screen. (**C**) Schematic of the DA9 motor neuron used to assess presynaptic assembly phenotypes.

The use of isPLA is relevant to multiple subcellular structures. In virology, isPLA has been used to study the interaction between viral capsids and the nuclear pore complex (NPC) during nuclear import. Studies on HIV-1 have used isPLA to illustrate the intimate association of the viral capsid with the NPC protein Nup358, an essential step for the virus to deliver its genome to the nucleus [85]. In neuroscience, isPLA has been adapted to image trans-synaptic complexes that span the synaptic cleft. By targeting a presynaptic protein, isPLA can generate a signal that reports an intact synapse (Figure 6), providing an extremely accurate method for measuring both the number and integrity of synapses [86].

### 3.4. Probing Dynamic Cellular Processes

Cellular processes are controlled by dynamic mechanisms involving the transient association and dissociation of protein complexes [87]. The ability of isPLA to capture these transient interactions in their physiological setting makes the technique well-suited to study such events.

Progression of the cell cycle is regulated by the ordered activation and inactivation of cyclin-dependent kinases (CDKs), which form transient complexes with their regulatory cyclin partners [88]. isPLA can be used to detect individual cyclin-CDK interactions at different stages of the cell cycle, providing an insight into a cell’s proliferative state. A study combining isPLA with flow cytometry showed that the cell cycle regulates the interaction between the CKS2 regulatory subunit and the mitochondrial protein SSBP1 in cervical cancer cells. In this study, Lyng et al. demonstrated that the levels of CKS2-SSBP1 PLA signals were at their peak during the G1 and early G2 phases by isPLA. This timing coincides with known mitochondrial DNA replication [89]. In another study, Neilsen showed that TGF-β-induced complex formation of Smad proteins was reduced in confluent cell cultures and differed by cell cycle stage, as detected by isPLA, indicating how cell density and cycle phase can impact signaling pathways [90].

Autophagy is a catabolic process of degrading and recycling cellular components that involves sequestering cytoplasmic cargo into double-membraned vesicles known as autophagosomes. Cargo recognition by autophagy receptors such as p62/SQSTM1 is a critical step, which connects the cargo to the autophagosome membrane protein LC3 [91]. isPLA is a powerful tool for tracking autophagic flux by directly visualizing the interaction between p62 and LC3. The increase in p62-LC3 PLA signals reports on autophagosome formation and cargo delivery for degradation. The method has several advantages over conventional approaches, isPLA measures endogenous protein interactions and directly reports on the cargo-loading step [92]. In addition, isPLA can be employed to investigate selective autophagy pathways. By targeting the mitochondrial receptor NIX and LC3, isPLA can specifically report on mitophagy, the selective degradation of mitochondria, offering a tool to explore the molecular machinery of individual autophagic pathways [93].

## 4. Translational and Clinical Applications of PLA

### 4.1. Dissecting Cancer Pathways and Identifying Biomarkers

In the field of cancer research, PLA is moving from a simple investigative tool to an overall clinical and translational platform. Through enabling the in situ visualization and quantitation of in situ protein signaling complexes within formalin-fixed paraffin-embedded (FFPE) tissues taken from patients, isPLA provides functional information that is often more effective than conventional immunohistochemistry (IHC) assays [39]. Traditional cancer biomarkers typically rely on measuring overexpression of individual proteins, such as the human epidermal growth factor receptor 2 (HER2) in the treatment of breast cancer [94]. However, protein expression levels do not necessarily reflect the status of biological pathway activation because receptors may exist in excess without having a true measure of functional activity. Through examining the protomer formation of individual oncogenic protein complexes, the isPLA facilitates a more direct and mechanism-relevant measure of pathway activity. Such measures are expected to reflect more accurately a tumor’s responsiveness to targeted therapies.

The HER2 tyrosine kinase receptor is an important mediator and target for therapy in breast cancer cases [94]. The oncogenic signaling of this receptor is activated through the heterodimerization of its subunits with other members of the same family, and through the formation of homodimers (HER2-HER2). Several studies suggest that the expression of HER2-HER3 heterodimers is closely correlated with aggressive clinicopathological features and represents an independent prognostic indicator of poor patient prognosis, featuring low relapse and overall survival rates [95,96,97]. This information is of clinical relevance, in that therapies like pertuzumab are engineered to inhibit the dimerization of HER2 [98]. Research into HER2 dimerization has employed isPLA. Interaction of HER2 with the cell adhesion molecule CEACAM6, measured by isPLA in tumor tissues, has been found to represent a robust predictive biomarker of the efficacy of treatment with trastuzumab. This result has the potential to act as a valuable companion diagnostic to aid treatment decisions [99].

**Figure 7:**
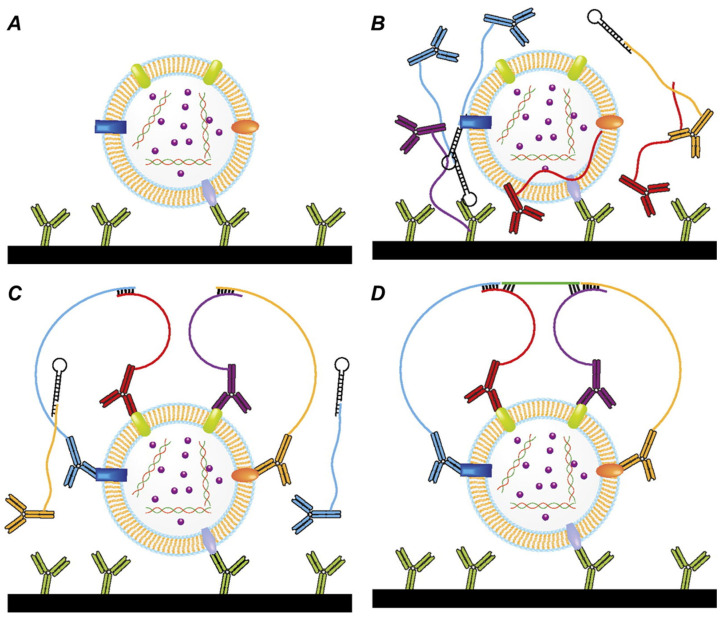
Mechanism of 4PLA Reprinted/adapted with permission from Ref. [101]. Copyright 2011, National Academy of Sciences. (**A**) Target molecules are captured by antibodies immobilized on the walls of a reaction vessel, (**B**) the four PLA probes are added, and the probes are allowed to bind different epitopes on the target structure. (**C**) The four oligonucleotides attached to the antibodies hybridize to each other and (**D**) guide hybridization of a further oligonucleotide.

Distinct from isPLA, another format of the PLA technology, solution-phase PLA has been utilized to detect new biomarkers from liquid biopsies. Prostasomes secreted by prostate epithelial cells into extracellular vesicles are found to be present in the blood plasma [100]. Moghaddam et al. established a highly specific 4PLA protocol that requires the simultaneous binding of five antibodies to four individual proteins situated on the outside surface of prostasomes (Figure 7). This ultra-sensitive assay demonstrated substantially elevated levels of circulating prostasomes in the plasma of cancer patients when comparing them to cancer-free individuals. It was further observed to quantify prostasomes levels correlating with the aggressive potential of tumors, represented by the Gleason score. Accordingly, prostasome measurement by 4PLA is a new blood-based biomarker for prostate cancer diagnosis and prognosis [101].

### 4.2. Unraveling Protein Pathologies in Neuroscience

A hallmark of neurodegenerative diseases is protein aggregation, inclusive of AD and PD [102]. The ultimate neurotoxic species are not the well-known, large, insoluble tangles and plaques that are classical markers of pathology but rather the more innocuous-looking small, oligomeric intermediate assemblies formed upon aggregation [103,104]. One of the major analytical hurdles is to detect these low-abundance, conformationally unique oligomers without geminal dilution and without confusing them with the much more numerous, non-toxic monomers found in patient tissues. As many closely spaced binding events are necessitated in the assay, PLA can be utilized to selectivity detect repetitive epitopes found within an aggregated conformation of a protein.

Generation of the amyloid-β (Aβ) peptide is an essential mechanism in Alzheimer’s disease (AD) pathogenesis [105]. To effectively detect soluble Aβ aggregates, Landegren et al. developed a solid-phase PLA that took advantage of the monoclonal antibody mAb158. This specific antibody shows selective affinity for a conformational epitope exposed on Aβ oligomers and protofibrils. The assay uses the same antibody as both capture and proximity probes; therefore, a signal is only generated if several mAb158 epitopes are proximal in space, suggesting the presence of an aggregate. The assay was shown to be extremely sensitive, with a limit of detection (LOD) of 0.1 pg/mL, and was able to detect Aβ protofibrils in the face of a 2.2-million-fold molar excess of monomeric Aβ. In addition, this assay was used to detect soluble Aβ aggregates in brain homogenates from transgenic AD model organisms [26]. PLA has also been used to detect tau pathology. In post-mortem human brains afflicted with AD, PLA was used to visualize and quantify aggregates of ubiquitin-modified, hyperphosphorylated tau, thus providing in situ demonstration of the specific PTMs associated with pathological tau aggregates [106].

The pathological feature of PD is the aggregation of the α-synuclein protein inside cells, which accumulate to give Lewy bodies [107]. Cappelletti et al. developed an α-synuclein PLA (AS-PLA) designed to detect the postulated toxic oligomeric precursors of the inclusions detected in paraffin-embedded sections of cerebral tissue. AS-PLA demonstrated a previously unappreciated topology of widespread α-synuclein oligomeric pathology that appears to arise before the classic formation of Lewy bodies. This finding supports the postulate that smaller oligomeric complexes contribute to the mechanism of the disease and highlights the unique ability of PLA to detect subtle pathological alterations [108].

### 4.3. Pathogen Detection and Infectious Disease Diagnostics

The selective and fast diagnosis of infectious diseases is important to treatment of individuals, control of epidemics, and safeguarding of public health. PLA is a technological platform suitable for developing ultrasensitive diagnostic tests. Through the combination of antibody-based recognition with nucleic acid amplifications, PLA is potentially able to acquire sensitivities orders of magnitude greater than standard antigen assays such as the lateral flow assay or the ELISA. Sensitivity renders the technology ideal for detection of infection at early time points when the pathogen burden is low [109].

Human immunodeficiency virus (HIV) cure strategies require sensitive assays for the detection and characterization of the viral reservoir. PLA has been applied to probe brain tissue from HIV-infected individuals to identify the viral capsid protein p24. Ma et al. detected latent HIV-1 DNA in perivascular macrophages and microglia. Since no p24 protein was detected, these cells did not appear to be utilized for producing new viruses. However, experimental results indicate that the virus’s genetic material was actually concealed within them. This suggests that the brain may serve as a significant latent reservoir for HIV [110]. PLA was also used in the discovery of molecular processes of viral replication. HIV-1 p6 Gag protein interactions with the host protein Tsg101 are a criterion process of viral egress [111]. PLA studies showed that an HIV-1 subtype C p6 Gag PTAP motif duplication characteristic of high viral loads significantly accelerates Gag-Tsg101 interaction compared to subtype B viruses without the duplication. The discovery offered a molecular-level explanation for why this dominant HIV-1 subtype replicated more efficiently [112].

**Figure 8:**
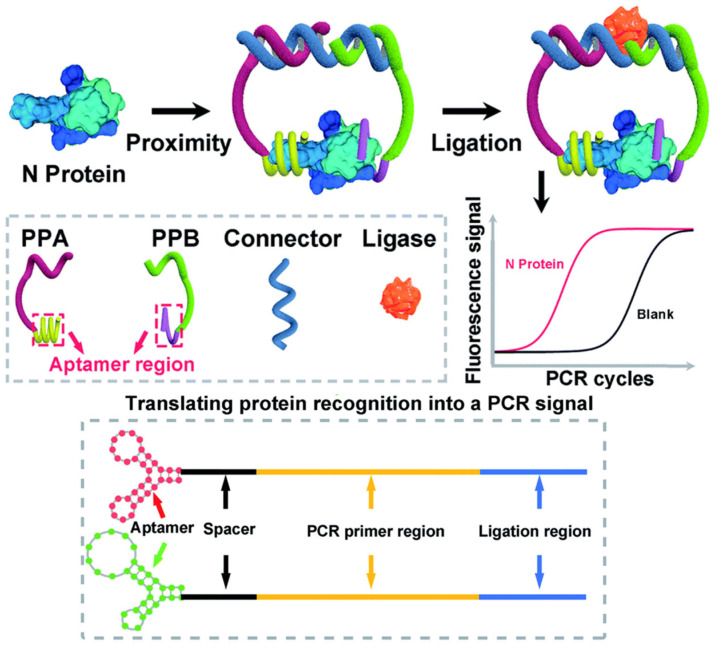
Scheme of aptamer-assisted PLA for COVID-19 antigens. Reprinted/adapted with permission from Ref. [115]. Copyright 2020, Royal Society of Chemistry.

The COVID-19 pandemic emphasized the requirement for fast and highly sensitive diagnostic tools for early detection of viral antigens [113]. Current rapid antigen assays, largely founded on lateral flow immunoassays, are not as specific as nucleic acid amplification tests (NAATs), with early false negative results during infection when the viral load is low [114]. The signal amplification of PLA could be utilized to design next-generation antigen detection assays for respiratory viruses. PLA could detect viral proteins, such as the SARS-CoV-2 spike or nucleocapsid protein, with sensitivity comparable to NAATs but with the benefit of an antigen test. Zhang et al. developed an aptamer-based PLA for the specific and highly sensitive detection of antigens associated with COVID-19 in serum samples (Figure 8). The principle of the assay involves the simultaneous binding of two distinct aptamer probes to a single protein target. This binding event brings the DNA oligonucleotides attached to each aptamer into close proximity, enabling their ligation. The resulting ligated DNA molecule then serves as a template for amplification by qPCR. Through this mechanism, the presence of a target protein is converted into a quantifiable DNA signal. This study showed the robustness of PLA by quantifying the concentration of serum nucleocapsid protein [115].

Malaria diagnosis in resource-constrained settings is nearly completely dependent on rapid diagnostic test (RDTs) to detect pLDH or HRP2 parasite antigens [116,117]. Up to a point, the sensitivity of the RDTs could fall below the cutoff to detect low-density parasitemia, a condition most typically found in asymptomatic carriers perpetuating ongoing transmission. PLA promises a new frontier of pLDH or HRP2 malaria diagnosis with vastly improved sensitivity. A PLA for pLDH or HRP2 could detect infections below the threshold of current RDTs. This capability will improve malaria elimination and case management by uncovering and treating the asymptomatic reservoir [118].

## 5. Technological Evolution and Advanced PLA Platforms

### 5.1. Enhancing Specificity and Sensitivity

Since the discovery of PLA, the fundamental technology has evolved substantially to deliver a better performance characteristic like specificity and sensitivity. These advances are realized through alterations about assay designs and innovations of proximity probes.

One of the earliest approaches to increasing specificity is raising the requirement for recognitions. Classical PLA is built upon binary recognition, multi-binder formats of PLA have been developed to differentiate extremely complex targets. A “3PLA” was conceived with three diverse antibody–oligonucleotide conjugates that must each bind to the same target to template formation of amplifiable DNA reporter. This approach was generalizable to a “4PLA” conceived to differentiate complex extracellular vesicles named prostasomes. This assay was engineered to include simultaneous binding of five antibodies, each to capture and four to proximity ligation, to form the PLA signal. Quadruple recognition requirement caused a remarkable 150-fold increase in a classical two-probe PLA’s sensitivity to the same target. Conclusion indicates that increasing recognition stringency is able to reduce background tremendously and increase detection limits [101].

**Figure 9:**
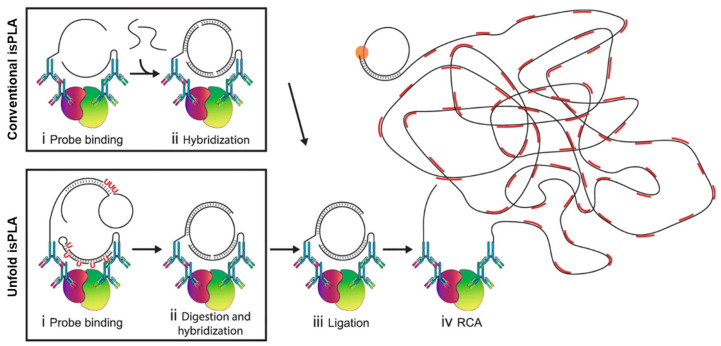
Schematic illustration of isPLA using conventional and UnFold probes. Reprinted/adapted with permission from Ref. [119]. Copyright 2018, Springer Nature.

Optimizations of oligonucleotide probe design have similarly enabled the introduction of substantial performance improvements. In the “UnFold probe” design, all DNA components for circle formation are contained within the two proximity probes, which removes the requirement for a separate connector oligonucleotide. One of the probes has a hairpin-loop oligonucleotide that, following enzymatic digestion, contributes the ends to ligation and the other holds the template sequence. The probes are inert and must get activated only after an enzymatic unfolding step is undergone following initial binding and washings. Therefore, the UnFold probe design holds back unbound probes from unwanted interactions before an appropriate time and creates an efficient ligation event to provide a stronger signal at a low concentration of probes when comparing to a traditional isPLA (Figure 9) [119]. Another novel format is the circular PLA (c-PLA), where two antibody oligonucleotides are employed to act in a guiding fashion for ligation of two free oligonucleotides to a circle [120]. This type of setup increases the stringency requirement, four fragments need to come together by chance to provide a background signal, and this makes c-PLA more reproducible and allows the use of low-affinity antibody.

### 5.2. Expanding Throughput

One of the principal limitations of early isPLA was low assay throughput, one assay normally only examined a single set of protein interactions at a time. Considerable efforts have gone into raising the assay throughput in order to permit more general and systematic investigations [121].

**Figure 10:**
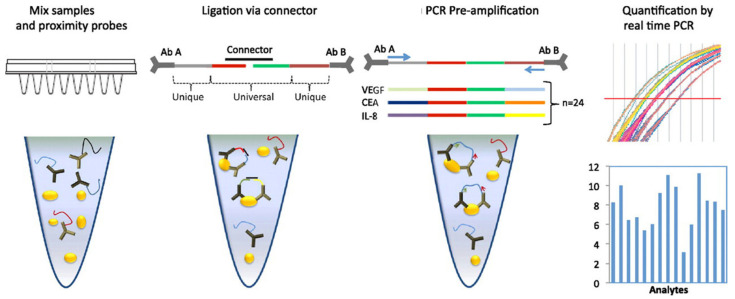
Schematic description of multiplex homogenous PLA Reprinted/adapted with permission from Ref. [33]. Copyright 2011, Elsevier.

Multiplexing is made possible through utilization of a particular set of primary antibodies in each of the interactions of interest. Each of the respective sets of PLA probes forms a unique DNA circle. Separate circles can be identified by oligonucleotides conjugated to unique fluorophores, such that numerous PPIs can be made detectable simultaneously from a single cell (Figure 10) [33].

A substantial throughput increase was obtained with the creation of high-throughput imaging PLA (HiPLA) that converts the isPLA protocol to a 384-well microplate platform. Misteli et al. described a HiPLA to study the interaction of 60 different nuclear proteins with the nuclear protein lamin A/C in a cellular model of Hutchinson-Gilford progeria syndrome. This HiPLA enabled the researchers to easily determine a subset of interactions uniquely altered by the disease-causing progerin mutation, and demonstrated the potential of HiPLA for broad-scale, image-based interactome mapping [122].

Furthermore, PLA in combination with microfluidics is another means to enhance throughput and experimental control. Microfluidic chips with hundreds of parallel cell culture chambers make the entire PLA pipeline automatable. Microfluidics provide a significant cost reduction in reagent usage, and precise spatiotemporal control of cell stimulation. Microfluidic-PLA enables dynamic signaling pathway profiling by measurement of multiple PTMs or PPIs on the same cells simultaneously within one experiment [77].

### 5.3. Integrating Advanced Readouts

With the incorporation of high-throughput analytical platforms such as next-generation sequencing (NGS) or mass cytometry (CyTOF) instead of traditional detection tools, PLA has been reformed from a targeted assay to different forms of omics discovery platforms, in order to construct spatially resolved interactome maps and single-cell proteomic profiling at deep levels [123].

In the proximity ligation sequencing assay (PLA-Seq), the imaging readout is replaced by sequencing readout [124]. Each proximity probe pair is engineered to produce a ligated DNA product with a specific barcode of the target protein. All DNA products of the subsequent PLA are pooled, amplified, and sequenced. Each of the sequences from unique barcodes in sequenced data corresponds to the frequency of the corresponding PPI. PLA-Seq is able to enumerate hundreds, even thousands of protein interactions in a single sample to generate an in situ protein interaction network [125]. Tay et al. developed a method called proximity sequencing that combines PLA with single-cell sequencing. The platform simultaneously measured extracellular proteins, protein complexes, and mRNAs in thousands of single cells, providing quadratically scaled multiplexing for detecting protein interactions. The application of this multiomic approach was expected to discover uncharacterized protein interactions and provided a deeper understanding of cellular processes, thereby enhancing single-cell phenotyping [126].

As a closely similar variant to PLA, the PEA technique can be integrated with NGS for high-throughput proteome-wide analysis. The PLA-Seq method is used to map thousands of PPIs and build in situ interactome networks. In contrast, PEA combined with sequencing is used to quantify thousands of proteins in bulk liquid samples, with a primary focus on determining biomarker abundance [127]. Lundberg et al. described a high-throughput method that combines PEA with automated sample preparation and a sequencing readout. The platform measured nearly 1500 proteins in 96 samples simultaneously, generating approximately 150,000 data points per run. The application of this platform was anticipated to significantly advance biomarker discovery for disease prediction and contributed to the fields of precision medicine and wellness monitoring [128].

**Figure 11:**
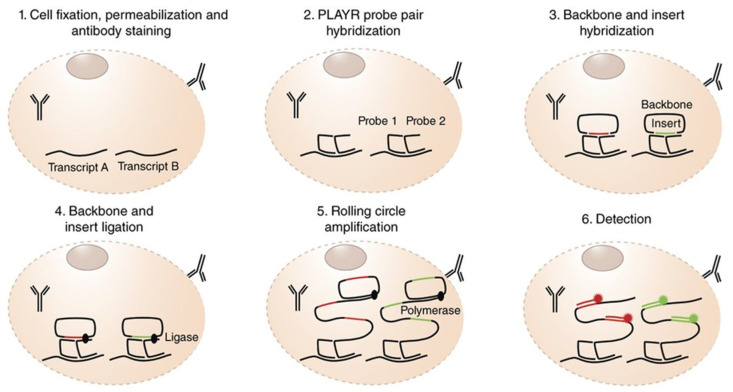
The main steps of the PLAYR protocol. Reprinted/adapted with permission from Ref. [131]. Copyright 2016, Springer Nature.

PLA can also be combined with CyTOF, a platform that utilizes heavy metal isotopes as reporters instead of fluorophores to move beyond the limitations of spectral overlap of fluorescence-based flow cytometry [129]. In PLA-CyTOF, terminal detection oligonucleotides are metal isotopically labeled. The CyTOF measures the cells gained from the isPLA reaction. It is possible for the CyTOF to measure over 40 diverse parameters per cell simultaneously. PLA-CyTOF was used to analyze large and diverse populations of cells. providing detailed information for each individual cell by performing two types of measurements at the same time. PLA-CyTOF used the PLA method to detect dozens of protein interactions and modifications. Concurrently, it usesd standard metal-tagged antibodies to measure the amounts of other proteins. The end result is high-dimensional, single-cell proteomic profiling correlating cell identity or cell cycle phase or signaling status to states of protein interactions within complex samples such as tissue dissociates or blood [130]. Gherardini et al. developed PLA for RNA (PLAYR) for highly multiplex transcript counting in individual cells (Figure 11). They applied PLAYR to primary cells, making use of an individual set of markers to define the identity and cell’s functional state and to measure another set of target transcripts. Adding RNA expression to high-throughput phenotyping, PLAYR developed a new metrology of describing cellular metabolism in detail [131].

For applications requiring portability and low cost, the PLA reaction can be combined with an electrochemical readout. In the electrochemical PLA (ECPLA) method, the conventional optical readout is replaced with an electrochemical signal. The proximity-dependent DNA ligation and amplification steps are coupled to an electrode-based detection system. A measurable change in current, potential, or impedance is typically generated by incorporating redox-active molecules or enzymes like horseradish peroxidase into the assay [15,132]. Primary advantages of ECPLA include high sensitivity, a wide dynamic detection range, and lower instrumentation costs compared to optical methods. Due to these advantages, the ECPLA method is highly suitable for the rapid and quantitative analysis of low-abundance biomarkers and for the development of portable biosensors and POCT devices [133]. Zhou et al. developed an enzyme-free electrochemical immunoassay for protein detection that uses palladium nanoparticles (PdNPs) as a signal label. In this assay, a catalyzed hairpin assembly (CHA) was initiated on an electrode surface by the binding of proximity probes to a target. The resulting long DNA structures then served as templates for the in situ synthesis of palladium nanoparticles (PdNPs) from palladium ions. The final electrochemical signal was generated by the efficient catalysis of NaBH_4_ oxidation by these newly formed PdNPs. When the method was applied to the detection of carcinoembryonic antigen (CEA), a LOD of 0.52 × 10^−16^ g/mL was achieved after signal amplification [134].

### 5.4. Beyond Antibodies

In addition to the signal improvements mentioned above for enhancing the performance of PLA, the recognition molecules used in the assay can also be replaced. The majority of existing PLA applications are built upon standard monoclonal or polyclonal antibodies as an initial point of a starter affinity reagent [32]. However, accessibility and consistency of high-quality antibodies are a major hindrance. These challenges have spurred the development of PLA platforms that utilize alternative binding molecules.

The initial idea of PLA was based on DNA aptamers [36]. Aptamers are of a synthetic chemical origin, which guarantees high batch-to-batch reproducibility. Aptamers are further extremely stable, and oligonucleotide nature facilitates labeling in a precise and facile manner with the reporter DNA strands. As a result of these important merits, the use of aptamers with PLA as an affinity reagent is revisited once again by the scientific world [135,136].

Nanobodies consist of fragments of camelid heavy chain-only antibodies. They are small in size, highly stable, and can differentiate new epitopes unseen by regular antibodies [137,138,139]. Bramham et al. built a nano-body-based PLA to image endogenously generated oligomers of the activity-regulated cytoskeleton-associated (Arc) protein within neurons’ dendrites. Employing two structurally different nano-bodies, each binding to structurally defined Arc protein binding sites, the assay was able to selectively differentiate Arc-Arc complexes. Constitutive, uniform oligomers of Arc within unstimulated neurons were revealed by the results. It is one way to attain higher structural selectivity in PLA, one way to challenge discrete structures of proteins and complex assemblies in vivo, when one is using nanobodies of well-defined binding sites [68]. Yang et al. compared a newly developed branched proximity hybridization assay (bPHA) to the standard isPLA using a nanobody-based system targeting a GFP fusion protein. The bPHA signal showed a strong linear correlation with target expression (R^2^ = 0.82), while the standard PLA signal did not (R^2^ = 0.007). The linear amplification of bPHA provides a more reliable quantitative readout than the non-linear amplification of PLA. This work also highlighted that using smaller probes like nanobodies can improve the molecular resolution of proximity assays [140].

The comparison of these advanced PLA platforms is shown in Table 3.

## 6. Perspectives and Conclusions

In spite of the revolutionary power of the PLA, the process is limited and has imperfections to contend with when it comes to experimental design and interpretation of data. Some of the most prominent among them include false-positive generation of signal by proximity noise [141]. False positives can occur when two non-interacting proteins are overexpressed at very high levels in a confined cellular compartment. The proteins may then happen to co-localize randomly within the assay’s 40 nm detection radius. In such a scenario, the resulting signal does not indicate a biologically relevant interaction [142]. To determine biological relevance of a PLA signal detected, quantitative comparison of signal in experimental models to stringent control sets must be made. Also, because PLA has a digital output in terms of spot counts, it is not an easy process to convert the output to an absolute measure of protein complexes/cell. The efficiency of signal generation can be influenced by many factors, including antibody affinity, epitope accessibility, steric hindrance between antibody–probe complexes, and the efficiency of the enzymatic ligation and amplification steps [120]. Therefore, PLA is best used for relative quantification, not absolute quantification. The method is effective for comparing changes in PLA signals between different experimental conditions. However, it is not suitable for determining absolute stoichiometry [142]. Lastly, the multi-step protocol of PLA is cumbersome, time-consuming, and expensive, especially in high-throughput studies [122].

The future development of PLA will exceed current limitations and expand the reach of the technology in quantitative, dynamic, and spatially resolved systems biology. Merging of PLA with NGS and CyTOF will continue to progress, allowing for routine, simultaneous interrogation of hundreds to thousands of in situ protein interactions. This will build condition-specific cellular interaction networks and complete interpretation of how proteome reorganizes in the presence of stimuli or disease [143]. For spatial omics, PLA can serve as a central technology. Platforms in the future will integrate PLA with spatial transcriptomics and genomics. Researchers will map protein interaction networks, gene expression profiles, and genomic features in parallel within the same tissue section. Such multi-modal information will provide a more complete representation of cellular function and tissue architecture [144]. Moreover, dependency on classical antibodies will diminish in the face of expanded usage of engineered affinity reagents such as nanobodies and aptamers. These reagents will increase consistency and afford additional stability and more freedom in probe design. At the same time, continued development of fully automated, microfluidic-based PLA platforms will decrease variability, decrease reagent cost, and make the technology more suitable for high-end throughput. Clinically, application of PLA-based assays will allow the approaches to become more robust and standardized. Researchers will observe more PLA-based companion diagnostics to forecast patient response to targeted therapy in cancer treatment. In infectious and neurodegenerative diseases, the sensitivity of PLA will result in the development of new clinical assays for the detection of early disease from minimally invasive sources like blood.

In conclusion, PLA has developed from an original idea of early proteins detection to a highly versatile platform from modern cell biology and translational research. Future innovations will continue to be incorporated into PLA. These include advances in affinity reagents, signal amplification, and high-throughput readouts. Integrating these developments will enable a more complete understanding of the complex molecular interactions in biology. The continued optimization of PLA will therefore provide a deeper, more functional understanding of cellular processes. This understanding will have applications in both basic research and clinical medicine.

## Figures and Tables

**Table 1:** The comparison between isPLA and solution-phase PLA.

Parameter	isPLA	Solution-Phase PLA
Primary Goal	Localization, visualization, contextual analysis	Quantification, profiling, biomarker discovery
Sample Type	Fixed cells, cytospins, tissue sections	Biological fluids (plasma, serum, urine), cell lysates
Sample State	Spatially intact, morphologically preserved	Homogenized liquid
Key Ligation Product	Circular DNA template	Linear DNA reporter molecule
Amplification Method	RCA	PCR
Readout Instrument	Fluorescence/confocal microscope, flow cytometer	qPCR instrument, digital PCR system, DNA sequencer
Key Advantage	Provides spatial context and subcellular localization	High sensitivity, high throughput, and high multiplexing capability
Primary Application	Mechanistic cell biology, pathology, validating interactions in situ	Clinical proteomics, diagnostics, systems biology

**Table 2:** Comparative Analysis of Key PPI Methods.

Feature	isPLA	Co-IP	FRET	Y2H
Principle	Proximity-dependent DNA ligation and amplification	Antibody-based pulldown of protein complexes	Non-radiative energy transfer between fluorophores	Reconstitution of a transcription factor
Sample type	Fixed cells/tissues	Cell/tissue lysate	Live cells	Live yeast cells
Endogenous proteins	Yes	Yes	No (requires fusion tags)	No (requires fusion tags)
Spatial information	High (subcellular localization)	None (bulk lysate)	High (live-cell imaging)	Low (nuclear localization only)
Temporal resolution	Low (endpoint assay)	Low (endpoint assay)	High (real-time dynamics)	Low (endpoint assay)
Sensitivity	Very High (single-molecule)	Moderate to Low	Moderate	High (genetic amplification)
Throughput	Low to Medium (HiPLA)	Low	Low	Very High (screening)
Primary use	In situ validation, localization	Biochemical validation, discovery (with MS)	Live-cell dynamics, distance measurement	Discovery screening
Key advantage	In situ detection of endogenous interactions with high sensitivity and spatial context	Gold standard for biochemical validation; can identify unknown partners	Real-time analysis in living cells with high spatial resolution	Unbiased, genome-wide screening for novel interactions
Key limitation	Endpoint assay; semi-quantitative; requires specific antibodies; risk of proximity artifacts	No spatial information; may miss transient interactions	Requires overexpression of fusion proteins; complex setup	High false-positive rate; non-physiological context

**Table 3:** Comparison of Advanced PLA Platforms.

Platform	Conventional isPLA	HiPLA	PLA-CyTOF	PLA-Seq	PLA with Super-Resolution	ECPLA
Primary readout	Fluorescent spots	Fluorescent spots (High-Content Imaging)	Fluorescence/Isotope signals (Single-cell)	DNA sequences (NGS)	Fluorescent spots (STED/STORM)	Electrical signal (Amperometr)
Throughput	Low	High	Very High	High	Very Low	High
Multiplexing capacity	Low (1–4 plex)	Low (1–2 plex per screen)	Medium to High (3–50+ plex)	Very High (100 s–1000 s+ plex)	Low (1–2 plex)	Low to Medium
Resolution	Diffraction-limited (~1 µm signal)	Diffraction-limited (~1 µm signal)	Single-cell population	Bulk tissue/cell population	Nanoscale (~20–50 nm)	Bulk sample
Primary advantage	Preserves subcellular spatial context for targeted interactions.	Enables systematic, image-based screening of interactomes.	High-dimensional, single-cell quantification of interactions and protein markers.	Enables discovery-oriented, interactome-scale profiling.	Visualizes the nanoscale organization of protein complexes in situ.	Low-cost, rapid, and portable; suitable for point-of-care diagnostics.
Key limitation	Low throughput; limited multiplexing.	Indirect multiplexing; requires large antibody libraries.	Loss of tissue architecture and subcellular spatial information.	Loss of single-cell and spatial resolution; complex bioinformatics.	Extremely low throughput; requires specialized microscopy.	Typically, lower multiplexing; less established for in situ use.

## Data Availability

The data that support the findings of this review study are openly available and collected from the published research articles, review papers with permission of publishers.

## Abbreviations

The following abbreviations are used in this manuscript:

AD	Alzheimer’s Disease
AP-MS	Affinity Purification-Mass Spectrometry
Aβ	Amyloid-β
BiFC	Bimolecular Fluorescence Complementation
CDKs	Cyclin-Dependent Kinases
CEA	Carcinoembryonic Antigen
CHA	Catalyzed Hairpin Assembly
Co-IP	Co-Immunoprecipitation
ECPLA	Electrochemical Proximity Ligation Assay
ELISA	Enzyme-Linked Immunosorbent Assay
ER	Endoplasmic Reticulum
FRET	Förster Resonance Energy Transfer
HER2	Human Epidermal growth factor Receptor 2
HIV	Human Immunodeficiency Virus
HRP2	Histidine-Rich Protein 2
IF	Immunofluorescence
IHC	Immunohistochemistry
isPLA	In situ Proximity Ligation Assay
LOD	Limit of Detection
MCS	Membrane Contact Sites
NAATs	Nucleic Acid Amplification Tests
NGS	Next-Generation Sequencing
NPC	Nuclear Pore Complex
PCR	Polymerase Chain Reaction
PD	Parkinson’s Disease
PDGF	Platelet-Derived Growth Factor
PdNPs	Palladium Nanoparticles
PEA	Proximity Extension Assay
PLA	Proximity Ligation Assay
PPIs	Protein–Protein Interactions
PTMs	Post-Translational Modifications
RCA	Rolling Circle Amplification
RDTs	Rapid Diagnostic Tests
RTKs	Receptor Tyrosine Kinases
Y2H	Yeast two-Hybrid
